# Characterizing Creative Thinking and Creative Achievements in Relation to Symptoms of Attention-Deficit/Hyperactivity Disorder and Autism Spectrum Disorder

**DOI:** 10.3389/fpsyt.2022.909202

**Published:** 2022-07-01

**Authors:** Marije Stolte, Victoria Trindade-Pons, Priscilla Vlaming, Babette Jakobi, Barbara Franke, Evelyn H. Kroesbergen, Matthijs Baas, Martine Hoogman

**Affiliations:** ^1^Educational Consultancy and Professional Development, Faculty of Social Sciences, Utrecht University, Utrecht, Netherlands; ^2^Department of Human Genetics, Radboud University Medical Center, Donders Institute for Brain, Cognition and Behavior, Radboud University Nijmegen, Nijmegen, Netherlands; ^3^Department of Psychiatry, Radboud University Medical Center, Donders Institute for Brain, Cognition and Behavior, Radboud University Nijmegen, Nijmegen, Netherlands; ^4^Department of Psychiatry and Department of Human Genetics, Radboud University Medical Center, Donders Institute for Brain, Cognition and Behavior, Radboud University Nijmegen, Nijmegen, Netherlands; ^5^Behavioral Science Institute, Radboud University, Nijmegen, Netherlands; ^6^Department of Psychology, University of Amsterdam, Amsterdam, Netherlands

**Keywords:** creativity, ADHD, ASD, neurodevelopmental disorders (NDDs), divergent thinking, convergent thinking, Creative Achievement Questionnaire (CAQ)

## Abstract

Previous research on ADHD and ASD has mainly focused on the deficits associated with these conditions, but there is also evidence for strengths. Unfortunately, our understanding of potential strengths in neurodevelopmental conditions is limited. One particular strength, creativity, has been associated with both ADHD and ASD. However, the distinct presentations of both conditions beg the question whether ADHD and ASD associate with the same or different aspects of creativity. Therefore, the current study investigated the links between ADHD and ASD symptoms, creative thinking abilities, and creative achievements. To investigate the spectrum of ADHD and ASD symptoms, self-reported ADHD and ASD symptoms, convergent (Remote Associations Test) and divergent thinking (Alternative Uses Task) and creative achievements (Creative Achievement Questionnaire) were assessed in a self-reportedly healthy sample of adults (*n* = 470). We performed correlation analysis to investigate the relation between ADHD/ASD symptoms and creativity measures. In a second phase of analysis, data from an adult ADHD case-control study (*n* = 151) were added to investigate the association between ADHD symptoms and divergent thinking in individuals with and without a diagnosis of ADHD.

Our analysis revealed that having more ADHD symptoms in the general population was associated with higher scores on all the outcome measures for divergent thinking (fluency, flexibility, and originality), but not for convergent thinking. Individuals with an ADHD diagnosis in the case-control sample also scored higher on measures of divergent thinking. Combining data of the population based and case-control studies showed that ADHD symptoms predict divergent thinking up to a certain level of symptoms. No significant associations were found between the total number of ASD symptoms and any of the creativity measures. However, explorative analyses showed interesting links between the ASD subdomains of problems with imagination and symptoms that relate to social difficulties. Our findings showed a link between ADHD symptoms and divergent thinking abilities that plateaus in the clinical spectrum of symptoms. For ASD symptoms, no relation was found with creativity measures. Increasing the knowledge about positive phenotypes associated with neurodevelopmental conditions and their symptom dimensions might aid psychoeducation, decrease stigmatization and improve quality of life of individuals living with such conditions.

## Introduction

Attention-deficit/hyperactivity disorder (ADHD) and autism spectrum disorder (ASD) are among the most common neurodevelopmental conditions. ADHD has a prevalence of around 5–7.8% in childhood and 1.2–7.3% in adulthood and ASD has a prevalence of around 1–2.8% in childhood and 2.5–3.4% in adulthood (1–9) with the comorbidity between the conditions reported as high as 68% in ASD (10, 11) and 12.4% in ADHD (12, 13). ADHD is currently defined by problems with sustained attention and/or hyperactive and impulsive behavior (14). ASD is characterized by deficits in social communication, sensory abnormalities, and restrictive repetitive behaviors (14). Symptoms of ADHD and ASD are dimensionally distributed in the general population (15). For instance, family members of individuals with ADHD and ASD often display subclinical symptoms of the condition (16, 17), and the neurobiology of ADHD traits in the population shows a large overlap with the neurobiology of being diagnosed with ADHD (18, 19). In addition, results show that subthreshold levels of symptoms can have an impact on important aspects of daily life such as employment and well–being (17). Therefore, ADHD and ASD symptoms concern a range of characteristics that individuals possess to a greater or lesser extent. While there is a fairly arbitrary cut-off point for a clinical diagnosis, this does not mean that there are no individuals with certain ADHD/ASD characteristics present in the general population. Diverse cognitive deficits are found associated with ADHD and ASD, most common are difficulties related to motivation and reward sensitivity, and issues with timing and executive dysfunctions in individuals with ADHD (20), and problems with the perception and processing of emotions, deficits in processing speed, theory of mind, and verbal learning and memory in individuals with clinical levels of ASD (21). Based on their deficit-related phenotypes, people with ADHD or ASD experience stigmatization, lower quality of life, and lower self-esteem (22–26).

Besides negative consequences, ADHD and ASD diagnosis and associated symptoms may not only lead to impairments alone but there might also be associated behavioral and/or cognitive strengths. Therefore, recent qualitative studies have examined self-reported strengths of ADHD and ASD (27–29). Examples of these self-reported strengths in ADHD include hyperfocus, divergent thinking, non-conformism, high energy levels, creativity, and empathy. It should be noted that while the associated strengths reported by these studies are likely to be due to ADHD symptomology and/or due to living with ADHD, possible use of medication to treat ADHD symptoms could be a source of variability. The positive traits associated with ASD are persistence, imagination, creativity, hyperfocus, increased cognitive functioning (memory and intelligence), and attention to detail (28–30). Creativity is reported as a strength linked to both conditions; also, a number of the other strengths have been linked to creative potential (31–34). Therefore, despite the differences in symptoms of ADHD and ASD, creativity is a promising candidate to be further evaluated in both these neurodevelopmental conditions (35).

Creativity is a broad concept. It can be defined by inventiveness and originality (36) resulting in the generation of new ideas or novel connections between constructs (37) that are useful within a certain social context (36). Creativity is a valued ability that is thought to be the driving force of discovery and innovation (38). For many years, theories of problem-solving divide creativity into the complementary concepts of divergent and convergent thinking (39, 40). Convergent thinking is defined as a focused and linear process of seeking one answer that is most fitting or most original, divergent thinking is associated with flexibility and diversification, resulting in the generation of a wide variety of answers for an open-ended question. Under this theoretical framework, creative potential is mostly associated with divergent thinking (36). However, it seems that both types of thinking are necessary for the production of a creative outcome (40–42). That is, the beginning stages of creative problem-solving rely more on divergent thinking and the later stages where a decision needs to be made or the best solution has to be identified probably rely more on convergent thinking (43).

While creativity is a self-reported strength in both ADHD and ASD, the relation with the conditions might not be comparable. In fact, research on the different cognitive profiles of ADHD and ASD points to the possibility that the relation between ADHD and ASD symptoms with creativity may be more complex and non-linear than was previously assumed. An important aspect to be considered is that interindividual differences in cognitive profiles are associated with distinct creativity profiles (42). Cognitive profiles refer to the individual clusters of characteristics linked to differences in information processing, perceiving, thinking, problem-solving, and remembering (44). The construct of the cognitive profile is widely used to understand creativity because it assumes that particular patterns of information processing can influence how each individual approaches problem-solving situations. Therefore, it may be possible that a group of individuals will have a similar cognitive profile that more strongly supports convergent thinking, while the cognitive profile of another group of individuals might be associated with superior divergent thinking abilities.

For ADHD, there is more evidence for a link with stronger divergent thinking than with convergent thinking abilities (45). However, this link between ADHD and divergent thinking is far from clear. Some studies have reported ADHD to be positively associated with increased scores in divergent thinking scales in children (31, 46–51) and in adults (52–55). Other studies do not find this association, neither in children (45, 56–62) nor in adults (63–65). There is also a great variety in results when the different aspects of divergent thinking, such as fluency, flexibility, and originality, are evaluated. For example, in the first study of White and Shah, they found increased fluency, flexibility and originality, in their second study they found only increased originality and in their third study they found increased flexibility and originality (52–54). These discrepancies could depend on the different tasks they used in their studies. All combined, the picture is far from clear and, in addition, the quality of the studies is often low, e.g. limited statistical power (66). The divergence of results may be caused by false positive and/or false negative results of studies, but a possible alternative explanation for the existence of both positive and negative associations between divergent thinking and ADHD might be differences in study populations, which included self-reported ADHD symptoms in the population as well as study designs involving clinically diagnosed cases and controls. Thus, one might argue that, ADHD symptoms up to a certain level might be beneficial for creativity, individuals that meet the diagnostic criteria may have deficits that are so severe and are often accompanied with other cognitive deficits that may hinder divergent thinking and creative expression. This explanation was earlier suggested in a review on ADHD traits and their similarity to gifted and creative behaviors (67), in analogy with research findings for other psychiatric disorders, such as schizophrenia, suggesting that an optimum number of psychiatric symptoms exists for creativity (68). However, the existing evidence for this theory in ADHD is currently limited, as there are only three population-based studies published on the relation between ADHD symptoms and divergent thinking with conflicting results. A positive link was found between ADHD symptoms and divergent thinking in a study in children, but only for fluency and not for flexibility (62). In a large-scale study in university students, a positive link between ADHD symptoms and creative originality was found (55). The third study did not find a link between ADHD symptoms and divergent thinking (65). To increase our understanding of the link between ADHD symptoms and divergent thinking, additional population-based studies are needed.

Compared to the situation in ADHD, the cognitive profile of ASD might be more beneficial for tasks that require convergent thinking. The cognitive profile of individuals with ASD is characterized with superior local processing and attention to detail (69–71), poor emotion recognition (72), improved non-verbal skills (73, 74). Despite the scarcity of studies that have empirically investigated the association between ASD and creativity, a recent clinical study found evidence for convergent thinking to be enhanced in children with ASD (75). This study also reported that divergent thinking scores of individuals with ASD were lower compared to controls. Another study showed performance advantages on convergent thinking tasks being associated with autistic traits in a sample of adults (76). Moreover, more original responses but less responses overall have also been reported for individuals with increased symptoms of ASD (77). However, a study in which performance was measured on a task with both convergent and divergent thinking components, found that the ASD group performed worse than the typically developing group (78). Therefore, in line with results from earlier studies, it may be that ASD is less likely to be associated with divergent thinking, at least when it comes to quantitative measures of divergent thinking, (79, 80) and more likely with convergent thinking. These results are in line with theoretical assumptions which state that the increased attention to detail and a preference for local over global processing in ASD might be beneficial in convergent thinking tasks (30, 69, 70), however this approach may be detrimental for divergent thinking.

An optimal way to investigate whether ADHD and ASD are associated with distinct types of creative thinking is to study creative thinking and (symptoms of) ADHD and ASD in the same sample. Interindividual variability in severity and/or type of symptoms is often overlooked in traditional study designs comparing two categorical groups, which leads to an important loss of depth and detail in the data and subsequent results. Because ADHD and ASD symptoms are distributed in the general population in a continuous manner, a population sample allows for an investigation of the link between ADHD/ASD symptoms, creative thinking, and creative achievements in the same sample, also preventing interference of the deficits that are associated with a clinical diagnosis. Additionally, previous creativity research made use of a variety of tasks measuring different constructs and domains, which makes it hard to draw conclusions (81). In order to learn more about the connection between (symptoms of) ADHD/ASD and different aspects of creative thinking, it is important to examine the same sample tasks. In the current study we thus examined the relationship between ADHD and ASD symptoms and their subscales (e.g., inattention for ADHD and rigidity for ASD) with divergent and convergent thinking tasks in an adult population-based sample. To further support our findings, we also investigated if ADHD and ASD symptoms and subscales are related to self-reports of recognized creative achievements. Based on the information discussed above, we hypothesized that ADHD symptoms are associated with divergent thinking, while ASD symptoms are associated with convergent thinking abilities. Finally, we explored the theory on the differential relationship between ADHD and divergent thinking in individuals with a diagnosis of ADHD and in those scoring high on ADHD symptoms in the population, by combining population-based and case-control datasets and investigating the full distribution of ADHD symptoms in relation to divergent thinking.

## Materials and Methods

### Participants

The current study is based on data that were collected as part of the Brain Imaging Genetics (BIG) project (82, 83). The BIG study is a study of self-reported healthy individuals included into earlier imaging studies at the Donders Centre for Cognitive Neuroimaging. The study was approved by the medical ethical committee (CMO region Arnhem/Nijmegen, The Netherlands) and all participants provided written informed consent prior to participation. Participants in the BIG study were invited to do online testing consisting of cognitive tasks and questionnaires in various waves. The sample size for the current study depended on data availability, ranging from a sample size of 215 individuals for the combination of ADHD/ASD symptoms and creative performance data and a sample of 470 individuals for the combination of ADHD/ASD symptoms and creative achievements data. For additional information on the online testing procedures, please see the Supplementary Methods.

To deepen our understanding of the link between ADHD and divergent thinking, we used data from the IMpACT2-NL sample, an adult ADHD case-control sample (79 cases and 72 controls). In this study the same instruments as in the BIG study were used to assess creativity, but a different instrument was used to assess ADHD symptoms, see below. For a description of the IMpACT2-NL sample and the study procedures, please see the Supplementary Methods.

### ADHD Symptoms

In the BIG study, ADHD symptoms were assessed using the ADHD Self-report Questionnaire (84). This questionnaire consists of 23 questions related to the 18 ADHD symptoms discussed in the Diagnostic and Statistical Manual of Mental Disorders 4^th^ edition (DSM-IV) (14, 85). For each symptom, the possible answer options are “0 = never/rarely”, “1 = sometimes”, “2 = often”, “3 = very often”. The participant is asked to find the answer that best fitted their behavior of the past 6 months. A total score for ADHD symptoms was computed by adding up all individual scores on the 23 questions. Scores could range between 0 (no ADHD symptoms) to 69 (highest amount of ADHD symptoms). Separate scores for the subscales “Inattention symptoms” and “Hyperactive/Impulsive symptoms” were also calculated, with ranges of 0–33 and 0–36, respectively. This ADHD questionnaire has good external validity (high correlation with clinician rated symptoms) and internal consistency between 0.72 and 0.83 for different subscales (84). In the IMpACT2-NL study, the Diagnostic Interview for Adult ADHD (DIVA) was used. The DIVA is an interview that is conducted by a trained researcher. The DIVA reports a yes (score of 1) or a no (score of 0) for each of the 18 ADHD symptoms from the DSM and has a good concurrent validity between 0.54 and 0.72 (86). The variable that was used from the DIVA in the current study is the total number of ADHD DSM symptoms with a range of 0–18, corresponding to the 18 ADHD symptoms. To combine the BIG and IMpACT2-NL ADHD symptoms, the self-reported ADHD symptoms from the BIG study were recoded into the 18 ADHD DSM symptoms, where scores 0 and 1 were recoded into 0 (no), and scores 2 and 3 were recoded into 1 (yes). Adding these scores resulted in a potential range of 0–18 ADHD symptoms, similar to the IMpACT2-NL ADHD symptoms. For the individual 18 ADHD DSM items see the Supplementary Methods.

### ASD Symptoms

To assess ASD symptoms the participants filled out the AQ18 questionnaire (87). In short, this questionnaire consists of six items that are based on the DSM-IV section about ASD and 12 items from the AQ which originally contains 50 items (88). For all 18 items, participants were instructed to choose how well the statement applies to them by selecting one of the following answer options: “1 = definitely agree”, “2 = partially agree”, “3 = partially disagree” and “4 = definitely disagree”. For a total score, the items were summed with a range of 18 (no autistic traits) to 72 (highest number of autistic traits). In addition, the 18 items were divided into five subscales: “child behavior” (range 4–16), “rigidity” (range 4–16), “social difficulties” (range 3–12), “attention to detail” (range 3–12), and “problems with imagination” (range 2–8), for the individual items see the Supplementary Methods. The AQ18 questionnaire has a high discriminant validity and satisfactory to good internal consistency [correlations between 0.54 and 0.86; (62, 64)].

### Divergent Thinking

The Alternative Uses Task (AUT) was used to assess divergent thinking abilities (39). Participants were asked to generate as many new and original ways to use an item as possible in 3 min. The items used in this study were a “brick”, “newspaper” and “shoe”. To give an example, for “brick” alternative uses could be, *use as a bookstand*, or *use as a paperweight*. Responses were scored on three different outcome measures of divergent thinking: 1) *fluency* reflects the number of non-redundant responses, 2) *flexibility* reflects the number of conceptual categories the responses belong to (e.g., for the item “brick” there is the category “built with” and “throw”), and 3) *originality* reflects how novel and uncommon the ideas are (rated from 1 = not original at all, to 5 = very original). For each item, two trained coders (MB&MH) counted all ideas to determine fluency and coded all ideas to determine flexibility and originality. Cohen's Kappa was calculated per item (newspaper, shoe, brick) to assess the overlap between the category scores (flexibility) made by the two raters. The overlap between category scores (flexibility) of the two raters was sufficient (Cohen's kappa of 0.76–0.85 for the three items, respectively). Additionally, the intraclass correlation coefficient to compare the originality scores of the two raters for each individual idea was found to be sufficient (ICC of 0.69–0.78 for each item, please see Supplementary Table 1). For each item, the average score of the two trained coders was used to derive scores for fluency, flexibility, and originality. Fluency, flexibility and originality ratings were then averaged across the three items.

### Convergent Thinking

The Remotes Associates Test (RAT) was used to assess convergent thinking (89). This task consists of 30 items, in which participants have to come up with one word that connects all three given words. For example, one of the items contains the words “beans”, “break”, “black” where the correct answer is “*coffee*”. There is only one correct answer for the individual items. The total scores were calculated as the number of correctly solved items, with a range of 0 to 30. The RAT has been found to have good internal consistency (Cronbach's alpha = 0.85) and convergent validity with other convergent thinking tasks [*r* = 0.32–0.39; (90)].

### Creative Achievements

Creative achievements were measured with the Creative Achievement Questionnaire [CAQ; (91)]. This is a self-report questionnaire that assesses creative achievements across ten domains: visual arts, music, dance, architectural design, creative writing, humor, inventions, scientific discovery, theatre and film, and culinary arts. Each of the ten domains has eight ranked questions weighted with a score of 0–7 with 0 = “No achievement”, 1 = “Training”, and the remaining six scores of achievement (e.g., “*I have won a national prize in the field of x”*, please see the example in the Supplementary Methods). Total scores were computed by adding up all scales, with a range of 0 to 70. We further grouped the domains into three subscales, namely a subscale for science/inventions domains (scientific discovery, inventions, culinary art with range 0–21), a subscale for expressive domains (humor, creative writing and visual arts with range 0–21), and a subscale for performance domains (dance, music, theatre and film with range 0–28) (91).

### Statistical Analyses

To identify associations between symptoms of neurodevelopmental conditions and creative thinking, we performed partial correlation analyses separate for ADHD total scores and ASD total scores with convergent (RAT scores) and divergent thinking (fluency, flexibility and originality of the AUT) scores, controlling for age and sex. We used a M_eff_ correction to adjust for multiple testing. The M_eff_ takes into account the correlation structure of the variables in the model and calculates the effective number of independent variables (92). For our hypothesis on creative thinking, four variables were included in the M_eff_ calculation: AUT Originality, AUT Flexibility, and AUT Fluency scores, and RAT total scores. The effective number of independent variables is 3.35, leading to a corrected significance threshold of *p* =0.015. In our explorative analysis of the ADHD symptoms domains of “inattention” and “hyperactivity/impulsivity”, and the ASD symptom domains of “child behavior”, “rigidity”, “social difficulties”, “attention to detail”, and “imagination” we reported associations with creativity measurements that researched the level of nominal significance, *p* <0.05 and interpreted these results with caution.

To investigate if ASD/ADHD symptoms and subscales were related to creative achievements, we investigated partial correlations of ASD/ADHD total scores with scores on the creative achievements questionnaire (CAQ), controlling for age and sex. To determine the level of statistical significance, we included four variables in the M_eff_ calculation: CAQ total score, CAQ science/interventions score, CAQ expressive score, and CAQ performance score. The effective number of independent variables was found to be 3, leading to a corrected significance threshold of *p* = 0.017.

To increase our understanding of the suggested differential relationship between ADHD symptoms and divergent thinking in people with an ADHD diagnosis versus people without an ADHD diagnosis, we combined the BIG and IMpACT2 datasets to show the entire distribution of divergent thinking scores across the full continuum of ADHD symptoms, from none in participants from the BIG study and controls from the IMpACT2-NL study) to the highest scores in individuals with an ADHD diagnosis (from the IMpACT2-NL study. To find out if there is indeed an inverted u-shaped relationship between ADHD symptoms and divergent thinking, we fitted a linear model and a quadratic model, including age and sex, and compare the r-squared values using IBM SPSS statistics 25 in the combined sample. In addition, we performed linear regression analysis to provide the betas for the term “ADHD symptoms” in the linear model separate for individuals with and without a diagnosis. This was done to investigate potential opposite effects of ADHD symptoms in the prediction of divergent thinking scores. To identify possible effects of stimulant medication on divergent thinking performance, we performed regression analyses including age, sex and a dichotomous variable for current psychostimulant use in individuals with ADHD.

## Results

The demographic information of the BIG (population-based) study sample is displayed in Table 1, and the creativity scores and the ADHD and ASD symptom scores of the BIG study are displayed in Table 2. Overall, the scores on the three creativity tasks (AUT, RAT, CAQ) were all significantly correlated (Supplementary Table 2). The highest correlations were observed for the three scores that were derived from the AUT: fluency, flexibility, and originality (*r* =0.43 to 0.90). In comparison, the correlations between the different constructs of creativity (convergent thinking, divergent thinking, and creative achievements) were small but significant (*r* =0.15 to 0.22), indicating that they all are linked to the bigger concept of creativity.

**Table 1:** Demographics of the population-based sample (BIG study).

	**Sample** ***N* = 470**	Subsample^*^ ***N* = 215**	***p*-value**
Percentage male	41%	42%	0.87
Age in years (SD)	36.1 (16.4)	37.9 (16.7)	0.19

* *Due to the various waves of the BIG study there is a large sample with available CAQ data and psychiatric symptoms (n = 470) and a subsample with complete creativity data (AUT, RAT, CAQ) and psychiatric symptoms data (n = 215). The differences between the sample sizes is a result of drop-out over time. The samples do not differ in terms of age or sex distribution*.

**Table 2:** Overview of the creativity measurements and self-reported symptoms of neurodevelopmental conditions in the population-based sample (BIG study).

	**Creativity scores**	**Average (SD)**	**n**
Divergent thinking	AUT originality	1.8 (0.3)	215
	AUT flexibility	6.7 (2.8)	215
	AUT fluency	9.4 (4.4)	215
Convergent thinking	RAT scores	12.7 (4.6)	215
Creative achievements^*^	CAQ total	5.1 (4.5)	470
	CAQ science (science, inventions, culinary)	2.3 (2.8)	470
	CAQ expressive (humor, writing, visual arts)	1.5 (2.2)	470
	CAQ performance (dance, drama, music)	1.2 (1.7)	470
NDD symptoms	ADHD symptoms (23-item questionnaire)	16.7 (7.9)	470
	ASD symptoms (AQ18)	36.3 (6.7)	470

*Displayed are the average raw scores for the creativity measurements and for the ADHD, ASD self-report questionnaires. NDD, neurodevelopmental disorders/conditions*.

* *Architectural performance of the CAQ is not included in any of the subscales because in the factors structure of Carson et al., architectural performance did not load on any of the three factors*.

Before performing the correlation analyses, the divergent thinking scores (AUT originality, fluency, and flexibility), convergent thinking scores (RAT), and creative achievements (CAQ) were normalized using rank-based transformation based on Blom's formula (93) in order to improve model fit.

### Associations of ADHD and ASD Symptom Scores and Subscales With Convergent and Divergent Thinking

We found significant correlations in the BIG sample between the total number of ADHD symptoms in the population and all the variables of the divergent thinking task (AUT), *r* =0.17 to 0.22, *p* < 0.012 (for a detailed overview of these results, see Table 3). The direction of these correlations was positive, indicating that higher rates of ADHD symptoms were associated with higher divergent thinking scores. We did not find significant correlations between the total number of ADHD symptoms and convergent thinking scores (*p* = 0.25) or between ASD scores and either of the convergent or divergent creativity measures (*p* > 0.26).

**Table 3:** Correlations of ADHD & ASD total scores with convergent and divergent thinking scores in the BIG study (population-based sample).

**Creative thinking score**	**ADHD total scores**	**ASD total scores**
RAT total score	*r* =0.08 *p* = 0.25	*r* = 0.08 *p* = 0.26
AUT fluency	***r*** **=** **0.19** ***p*** **=** **0.005**	*r* = −0.004 *p* = 0.96
AUT flexibility	***r*** **=** **0.22** ***p*** **=** **0.001**	*r* = 0.04 *p* = 0.59
AUT originality	***r*** **=** **0.17** ***p*** **=** **0.012**	*r* = 0.003 *p* = 0.97

*Displayed are the correlation coefficients (r) and p–values for the partial correlations between ADHD/ASD total scores and convergent (RAT) and divergent thinking (AUT) scores in 215 adult subjects of the BIG/Cognomics sample. Values in bold represent results that are significant after correction for multiple testing*.

We explored if there were associations between the symptom subscales on ADHD and ASD and convergent or divergent thinking. In Table 4 the full correlation matrix of these explorative analyses is presented. For the two ADHD subscales, the results indicate that both the “inattention” subscale and the “hyperactive/impulsivity” subscale contributed to the association with the fluency and flexibility scores on the divergent thinking task. The symptom domain of “inattention” seemed the main contributor for the association with the originality scores on the divergent thinking task.

**Table 4:** Exploration of the link between ADHD & ASD symptom domains and creative thinking in the BIG study (population–based sample).

**Symptom domains**	**RAT total score**	**AUT fluency**	**AUT flexibility**	**AUT originality**
Inattention	*r* = 0.03, *p* = 0.64	***r*** **=0.16**, ***p*** **=** **0.02**	***r*** **=** **0.22**, ***p*** **=** **0.002**	***r*** **=** **0.21**, ***p*** **=** **0.002**
Hyperactivity/ Impulsivity	*r* = 0.11, *p* = 0.12	***r*** **=** **0.17**, ***p*** **=** **0.01**	***r*** **=** **0.17**, ***p*** **=** **0.02**	*r* = 0.09, *p* = 0.21
Child behavior	*r* = 0.05, *p* = 0.45	*r* = 0.09, *p* = 0.20	*r* = 0.09, *p* = 0.20	*r* = 0.09, *p* = 0.17
Rigidity	*r* = −0.01, *p* =0.88	*r* = −0.03, *p* =0.62	*r* = 0.02, *p* = 0.74	*r* = 0.03, *p* = 0.66
Social difficulties	*r* = 0.14, *p* = 0.04	*r* = 0.01, *p* = 0.97	*r* = 0.06, *p* = 0.39	*r* = −0.01, *p* = 0.93
Attention to detail	*r* = 0.11, *p* = 0.10	*r* = −0.05, *p* = 0.44	*r* =0.03, *p* = 0.64	*r* = −0.03, *p* = 0.64
Problems with imagination	*r* = −0.03, *p* = 0.68	*r* = −0.13, *p* = 0.06	*r* = −0.18, *p* = 0.01	*r* = −0.23, *p* <0.001

*Displayed are the correlation coefficients (r) and p–values for the partial correlations between ADHD/ASD subscale scores and creative thinking measures in 215 adult subjects of the BIG/Cognomics sample. Values in bold are nominal significant correlations (p < 0.05)*.

Although we found no significant associations between the total number of ASD symptoms and convergent or divergent thinking scores, taking a closer look at the ASD subscales revealed three interesting links with creativity. First, there was a nominal significant negative correlation between the subscale “problems with imagination” and flexibility of the AUT (*r* = −0.18, *p* = 0.01). Second, there was a nominal significant negative correlation between the subscale “problems with imagination” and originality of the AUT (*r* = −0.23, *p* < 0.001). The “problems with imagination” subscale includes items such as “*I find making up stories easy*” and “*as a child, I enjoyed playing games involving pretending with other children”*. Hence, according to these results, having more problems with imagination is associated with lower flexibility and originality. Third, a positive correlation between the subscale “social difficulties” and convergent thinking was found (*r* = 0.14, *p* = 0.04). This indicates that having less social skills/more social difficulties is associated with increased convergent thinking.

### Associations Between ADHD and ASD Symptom Scores and Subscales With Creative Achievements

The total number of ADHD symptoms showed a small nominal significant correlation with the total score of the CAQ (*r* = 0.10, *p* = 0.02, Table 5), which did not survive correction for multiple testing. This finding appeared to be explained by a positive correlation between the total number of ADHD symptoms and creative achievements in the subscale expressive creativity (i.e., humor, visual arts, and writing), which reached statistical significance, *r* = 0.15, *p* = 0.001.

**Table 5:** Correlations of ADHD and ASD total scores with creative achievement scores in the BIG study (population-based sample).

**Creative achievement score**	**ADHD total scores**	**ASD total scores**
CAQ total score	*r =* 0.10, *p* = 0.02	*r =* 0.03, *p* = 0.54
CAQ science subscale	*r =* 0.06, *p* = 0.23	*r =* 0.10, *p* = 0.03
CAQ expressive subscale	***r** **=*** **0.15**, ***p*** **=** **0.001**	*r =* 0.01, *p* = 0.90
CAQ performance subscale	*r =* 0.03, *p* = 0.48	*r =* −0.10, *p* = 0.03

*Displayed are the correlation coefficients (r) and p-values for the partial correlations between ADHD/ASD symptoms and creative achievement scores of the CAQ in 470 adult subjects of the BIG/Cognomics sample. Values in bold represent results that are significant after correction for multiple testing*.

For ASD, none of the correlations with any of the creative achievement domains survived correction for multiple testing (*p* > 0.03). There were, however, two correlations that reached nominal significance: a positive link was seen between ASD symptoms and creative achievements in the science/interventions subscale (*r* = 0.10 *p* = 0.03), with a role for the symptom domain of attention to detail (Supplementary Table 3) and a negative association between ASD symptoms and creative achievements in the performances subscale (i.e., dance, drama and music) (*r* = −0.10, *p* = 0.03), possibly due to limited imagination skills (Supplementary Table 3). For a complete overview, we provide the correlations between the ADHD/ASD symptom domains and CAQ scores in the supplement (Supplementary Table 3).

### Distribution of Divergent Thinking Scores Across the Entire ADHD Continuum

To learn more about the relationship between ADHD symptoms and divergent thinking we combined the population-based sample (BIG) with the IMpACT2 sample, a case-control study (for demographics see Supplementary Table 4). To provide a complete overview of our results we also provide statistics for the case-control comparisons on divergent thinking scores in this table. The case-control analyses showed that individuals with ADHD scored higher, on average, on fluency and flexibility of the AUT than controls (*p* <0.001) in the IMpACT2 study.

**Figure 1:**
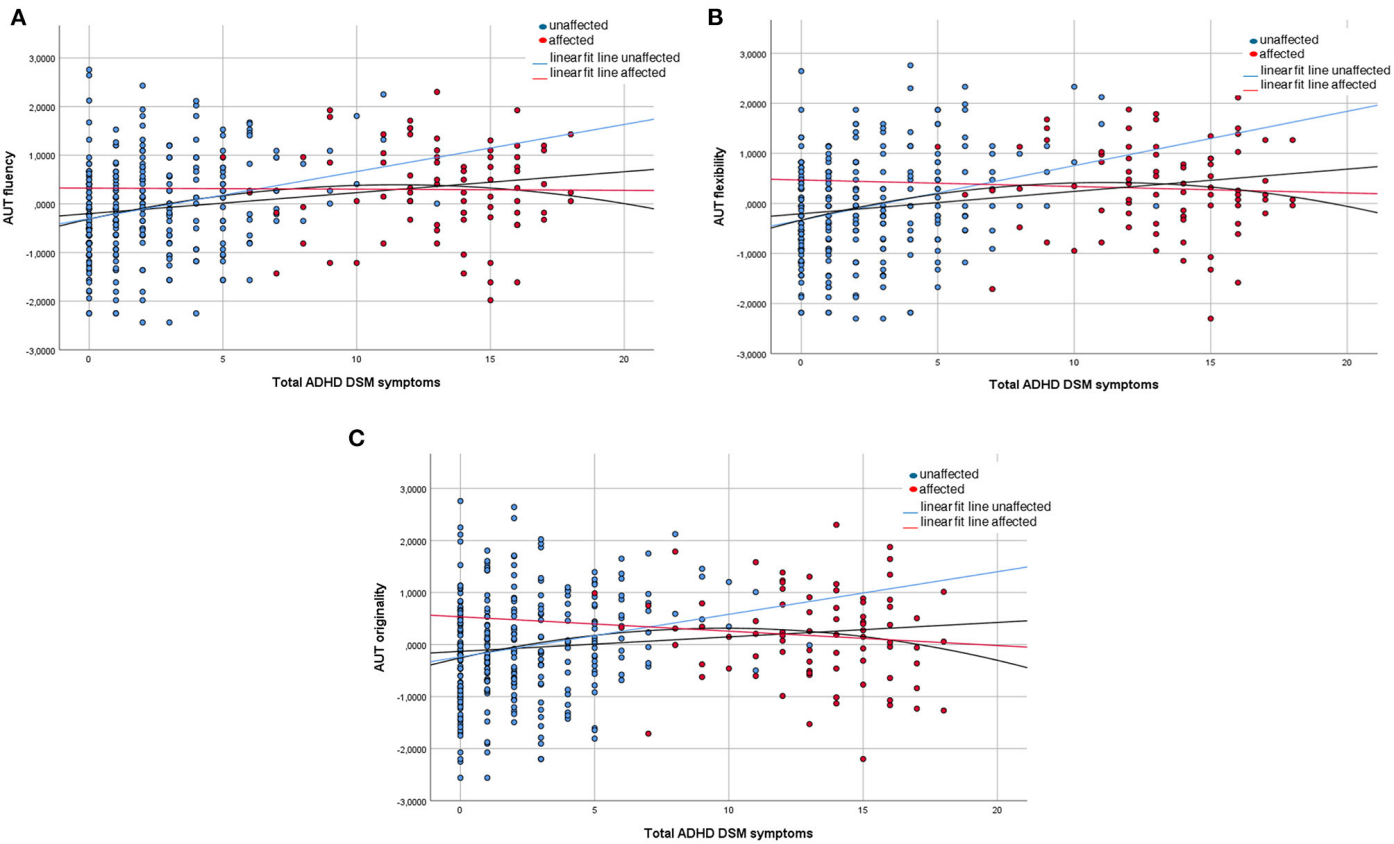
Divergent thinking across the ADHD continuum. Displayed are the distributions of the fluency **(A)**, flexibility **(B)**, and originality **(C)**, subscores of the Alternative Uses Task, across the ADHD continuum in 215 subjects of the BIG study and 151 (79 cases and 72 controls) subjects of the IMpACT2 study. The blue dots represent the scores of the individuals without a diagnosis, the red dots represent the scores of the individuals with a diagnosis. The dark lines represent the linear and quadratic fit of the model for the combined datasets, the red line is the linear fit for the individuals with a diagnosis, and the blue line is the linear fit for the individuals with a diagnosis.

In Figure 1 we display the number of ADHD symptoms and the divergent thinking scores for the population-based sample (BIG study) and the case-control sample (IMpACT2 study) combined. For fluency, flexibility, and originality, the quadratic models fitted better than the linear models, hinting towards an inverted u-shaped relationship between ADHD symptoms and divergent thinking. There might thus be an optimum level of ADHD symptoms for divergent thinking (Table 6). However, the R-squared values of the models are very low, explaining 2–7% of variance in the model, and therefore, we have to assume that there are other factors also involved in explaining divergent thinking scores.

**Table 6:** Overview of the output of the regression models for the relationship between divergent thinking and ADHD symptoms across the ADHD continuum.

	**R^**2**^**	***p*- value model**	**Standardized beta's ADHD symptoms**	***p*-value ADHD symptoms in the model**
**Fluency**				
Linear model combined data	0.08	<0.0001	0.21	<0.0001
Quadratic model combined data	0.09	<0.0001	0.58	0.001
Linear model individuals without diagnosis	0.09	<0.0001	0.22	<0.0001
Linear model individuals with diagnosis	0.01	0.82	−0.02	0.84
**Flexibility**				
Linear model combined data	0.10	<0.0001	0.21	<0.0001
Quadratic model combined data	0.12	<0.0001	0.63	<0.0001
Linear model individuals without diagnosis	0.13	<0.0001	0.24	<0.0001
Linear model individuals with diagnosis	0.008	0.90	−0.06	0.64
**Originality**				
Linear model combined data	0.04	0.001	0.13	0.015
Quadratic model combined data	0.06	<0.0001	0.55	0.003
Linear model individuals without diagnosis	0.08	<0.0001	0.18	0.003
Linear model individuals with diagnosis	0.03	0.58	−0.09	0.42

When we performed the linear regression analyses separate for individuals with and without a diagnosis, we found that the betas for the variable ADHD symptoms were positive and significant in the model in the group of individuals without a diagnosis (e.g., flexibility β = 0.24, *p* < 0.0,001, for more results, please see Table 6). However, in the group of individuals with a diagnosis, the betas for the variable ADHD symptoms are close to zero and are non-significant (e.g., flexibility β = −0.06, *p* = 0.64) in explaining divergent thinking. This means that, inindividuals with a diagnosis, the number of ADHD symptoms does not explain variance in divergent thinking scores. No effects of current psychostimulant medication use were found on divergent thinking (Supplementary Table 5).

## Discussion

In the current study, we aimed to shed a light on the link between symptoms of neurodevelopmental conditions, ADHD and ASD, and creative thinking abilities and creative achievements. The use of a population-based sample allowed us to investigate the link between ASD/ADHD symptoms and creativity without interference by the deficits that are associated with the more severe/clinical expression of the ADHD phenotypes. The results revealed that the total number of ADHD symptoms was related to higher divergent thinking scores of fluency, flexibility, and originality, but was unrelated to convergent thinking performance, confirming our hypothesis. The explorative analyses of individual ADHD domains showed that the inattention symptom domain was positively associated with all three divergent thinking outcomes whereas the symptom domain of hyperactivity/impulsivity was associated with fluency and flexibility. By combining a population-based sample and a case-control sample, we showed that ADHD symptoms predict divergent thinking up to a given symptom level at which the relationship plateaus. Our results did not confirm the hypothesis that ASD symptoms are related to increased convergent thinking. Instead, we found no significant correlations between ASD and any of the creativity measures in our main analyses. However, taking a closer look at the ASD symptom subdomains revealed that more imagination problems might be linked to less original and less flexible responses in the divergent thinking task and that more social difficulties seem to be associated with better convergent thinking. These explorative analyses have to be confirmed in future research.

Previous population based studies (55, 62) and a review on creativity in ADHD (66) indicated that there is a positive association between ADHD symptoms in the population and divergent thinking. The current results corroborate those previous findings and indicate that subclinical symptoms of ADHD are beneficial for divergent thinking. In combination with our explorative results that ADHD inattention symptoms were related to all divergent thinking outcomes, the current study strengthens the idea that inattention and behavior such as mind-wandering can facilitate divergent thinking and idea generation (94, 95). In general, being easily distracted is viewed as a negative characteristic of ADHD. However, it has been theorized that it also leads to the ability to perceive more and different external stimuli from the environment. According to the theory of situated creativity, the creative process is a cognitive process related to both the individual and its environment (96). Hence, it seems plausible that if a creative task is administered in a stimulus-rich environment, and the individual completing the task, is able to pick up on a variety of environmental cues, this combination could lead to more novel outcomes. This theory is supported by empirical evidence that a broad attentional focus, due to deficient latent inhibition (57), facilitates originality and flexibility (97). Furthermore, selective attention has been found to be negatively related to the generation of original ideas, although original ideas are more often formulated towards the end of a task (98). In other words, although it might take some time to come up with original responses, being slightly distracted might lead to noticing something that may seem irrelevant at first but can be incorporated into the creative task, thereby increasing originality (99, 100). This might also be an explanation as to why ADHD symptoms of hyperactivity/impulsivity were positively related to fluency and flexibility but not originality in the current study. One reason that originality was not associated with symptoms of increased hyperactivity/impulsivity might be that original answers are more likely to be generated when individuals have been working on a task for some time (78). According to the dual-pathway of creativity, there are two possible routes to creative responses. On the one hand, flexible processing of information can lead to creativity. Here, people can easily switch between different perspectives and tend to use a broad attentional style, like individuals with inattentive symptoms possibly do. On the other hand, task-persistence can lead to creativity by examining one perspective in depth and focusing on the details (101). This second route has been connected to continued effort and spending a longer time on divergent thinking tasks (102–104). While decreased attention presumably leads to more distractions by irrelevant stimuli in the environment due to deficient latent inhibition (57), individuals with symptoms of hyperactivity/impulsivity hypothetically do not reap the benefits of these distractions that can lead to more original responses because their hyperactivity/impulsivity lowers time on task (101).

In the current study we aspired to learn more about the complicated relationship between ADHD symptoms, ADHD diagnoses and divergent thinking. In previous research there have been studies that showed positive, negative and no effects for divergent thinking in case-control studies (66). In our case-control study we found individuals with an ADHD diagnosis to outperform, on average, controls on divergent thinking, especially fluency and flexibility. It has been hypothesized that increased levels of ADHD symptoms are beneficial for divergent thinking, but that in people with an ADHD diagnosis, associated deficits might mask or interfere with divergent thinking (67) leading to lower scores on divergent thinking tasks. Therefore, we combined population-based data and case-control data. The results for the combined data hint towards an inverted u-shaped relationship between ADHD symptoms and creativity as the quadratic fit of the prediction of ADHD symptoms and divergent thinking was superior to the linear fit. However, our data also showed that ADHD symptoms predicted divergent thinking up to a certain level of symptoms after which point the relationship plateaus. We suggest future research to cover the entire ADHD spectrum to examine whether divergent thinking breaks down at more extreme ADHD levels, which cannot be established with the current sample as we might not have the complete continuum of ADHD in our combined data set, possibly due to individuals that are severely affected by their ADHD symptoms not participating in research activities. Not finding sufficient evidence for a curvilinear relationship in our data also means that we cannot determine whether potential masking effects on divergent thinking are present in individuals at the extreme end of the distribution. In other words, it is possible that when ADHD symptoms are severe, they will overshadow the potential benefits (59). To learn more about this phenomenon, it would be helpful if future research would be aimed at identifying the cognitive deficits that are associated with divergent thinking: those cognitive deficits could then be directly investigated to learn more about potential masking effects on divergent thinking in individuals with ADHD. In addition, the notion of a potential inverted U-shaped relationship between ADHD and creativity is also interesting from a neurobiological perspective. It has been suggested that the purported mechanism is dopaminergic (105), which is interesting given the link between ADHD and dopamine. Future research into the relation between dopamine, creativity and ADHD could further delineate neurobiological mechanisms involved.

While we did find an association between ADHD symptoms and divergent thinking, ASD symptoms were not found related to convergent thinking or divergent thinking. This contrasts with a recent study with a clinical sample, which suggested that divergent thinking could be enhanced in ASD (75). Moreover, another study also reported associations between autistic traits and performance advantages on convergent thinking in a population-based sample (76). In both those previous studies different creative thinking tasks (a mathematical multiple solutions tasks and anagrams) were used. Based on these discrepancies, it seems that creative thinking abilities might only be related to symptoms of ASD when specific creativity domains are assessed. Convergent thinking as assessed with a mathematical task has been associated with ASD on several accounts (106, 107). These studies suggest that this domain fits the ASD cognitive profile best in their quest for order or reason (70). Domain-general creativity tasks, such as the RAT, might not fit the distinct strengths related to symptoms of ASD well enough. Therefore, we recommend future research to investigate the role of ASD and ADHD while taking domain-specificity into account and administering multiple convergent and divergent thinking tests that measure domains such as mathematics, arts, and science. Finding the overarching word that fits while performing the RAT can be seen as a global organization of material generated from the local elements of the task, which is hard for individuals with ASD because of their detail oriented cognitive style (108). Furthermore, the strong verbal component that is present in the RAT, our convergent thinking task, may not fit the cognitive profile associated with ASD. In general, ASD is associated with increased non-verbal abilities which suggests that a visual convergent thinking task may lead to different results (73, 74). Moreover, based on this theory, we suggest that future research investigates ADHD/ASD in relation to the influence of type of instruction and other task characteristics of creativity tasks. For instance, given the distinct cognitive profiles associated with ADHD and ASD, it might be possible to increase divergent thinking performance in individuals with ASD and convergent thinking performance in individuals with ADHD by providing specific task instructions directing them towards one or the other frame of thinking. Indeed, previous research reported that while individuals with ASD have a preference for local over global features, they are not incapable of conforming to instructions to focus more on global attributes (109).

While we could not identify a link between the complete construct of ASD and creativity, looking at different symptom domains revealed that more problems with imagination were related to lower divergent thinking scores, especially originality, and that having more social difficulties seemed to be associated with better convergent thinking. This second finding is in line with results from a meta-analysis that found that a certain degree of unconventionality and unconscientiousness seem to be associated with creativity (110). In the presence of more social problems, there might be more room for introspection, objective observations, drawing inferences, and task focused attention. More social problems could thus be linked to more focused attention, facilitating convergent thinking (110). Research generally reports that ASD is characterized by rigidity and typically associated with a lack of fantasy, imagination, and divergent thinking (79, 111). Moreover, pretend play, an important indicator of imagination in children and argued to be a precursor for adult creativity (112), is impaired in ASD (113). Early studies support the notion of impaired divergent thinking and imagination problems in individuals with ASD (79, 80). Hence, our results corroborate previous findings suggesting that being more imaginative facilitates divergent thinking.

Besides empirical measures of convergent and divergent thinking, we also examined the influence of ASD and ADHD on real world creative achievements. We found that individuals that reported more ADHD symptoms were more likely to report more expressive creative achievements (i.e. achievements in humor, creative writing, and visual arts) which is in line with previous research (65). Furthermore, a clinical study found that those with ADHD report more publicly recognized creative achievements (53). These findings suggest that even when symptoms warrant a clinical diagnosis, there is still the possibility of the strengths outshining the deficits. Previous findings suggest that symptoms related to hyperactivity and impulsivity are positive predictors of creative achievements but these achievements were not divided into domains or subscales (55). Therefore, future research is advised to take symptom subscale and creativity domain into account at the same time to understand which cognitive profile of ADHD and ASD fits which domain best. Additionally, we suggest to examine which regulatory mechanisms might influence creativity, once symptoms of ADHD are in the clinical range. For instance, having low self-esteem or feelings of incompetence might hinder creativity while higher intelligence and working memory capacity might help creativity in these cases (114–116). Meanwhile, ASD symptoms were not related to creative achievements at all. A reason for this may be that both convergent and divergent abilities are necessary to attain such achievements (76, 117), with divergent abilities being the strongest predictor of the two (118).In addition, since the CAQ is a self-report measure, it is possible that individuals with more symptoms underreported creative achievements due to the believed decreased levels of self-esteem in these populations (22–24).

The current study is the first to examine both ADHD and ASD symptoms and their relation to several measures of creativity in the same population sample. This allowed us to compare symptoms of ADHD and ASD on multiple aspects of creativity. In addition, the large population sample made it possible to investigate if distinct symptom profiles related to ADHD or ASD were associated with specific types of creativity. However, the current study also holds several limitations that should be taken into account. First, the data were collected as part of multiple waves of testing and therefore various measures were collected at different time points which may have influenced our results. Second, while it is commendable to be able to report on three different constructs of creativity (i.e., divergent thinking, convergent thinking, and creative achievements in daily life) it is important to note that results can deviate based on the creativity domain that is tapped by different tests (119, 120). In addition, we averaged the originality score across the three items of the Alternative Uses Task, as this was also done in previous studies, but this might result in the very original scores to be overshadowed by less original ones, making interpretation challenging. A final limitation could be that we combined the population-based and the case-control datasets although the ADHD instrument that was used in the two different studies was not the same (the creativity measures were). As previous research has shown a high correspondence between observed and self-rating of ADHD symptoms (121), we expected the impact of using these different instruments to be minimal. Finally, in examining the overlap and differences in ADHD and ASD in relation to creativity, it would be of interest in future studies to include people with diagnosed ASD in the analysis, as these were not available in the current study. Given the high comorbidity between both conditions [e.g., (10) and some of the neurobiological aspects that overlap in ADHD and ASD (122, 123)], including individuals with diagnosed ASD would provide a more complete picture.

To conclude, the neurodevelopmental conditions and associated symptoms of ADHD and ASD are well known for the difficulties they cause in functioning in daily life as expected by modern society. Individuals with ADHD or ASD might experience difficulties in school, work, and in their relationships (124, 125). Due to these problems, there is reduced quality of life for individuals with ADHD or ASD, and increased societal costs, such as increased health care related costs and losses due to absence or reduced productivity at work (22, 126). The current study shows that, next to the difficulties, there can also be strengths that accompany having (symptoms of) ADHD or ASD. Unraveling strengths of neurodevelopmental conditions and learning more about how individuals with (symptoms of) ASD and ADHD process information and perform cognitive tasks can lead to insights into underlying mechanisms of the conditions. Eventually, it can lead to novel intervention approaches and customization of educational programs to increase the chances that individuals with ADHD and ASD are and will remain assets instead of a financial burden to society due to school dropout and longer study duration. Creativity is one of the key abilities to thrive and solve the problems of today's complex society (127, 128). Putting more emphasis on the strengths of neurodivergent individuals will increase their well-being, reduce stigmatization, and therefore improve their quality of life because creativity can be an outlet for emotions, a source of pride or even a source of income. It will also benefit society as a whole to move away from looking at the neurodevelopmental deficit model and move towards the neurodivergent perspective (129).

## Data Availability Statement

The datasets presented in this article are not readily available because conditions for data use apply. Requests to access the datasets should be directed to https://www.impactadhdgenomics.com/ and/or https://www.ru.nl/donders/research/research-facilities-projects/cognomics/.

## Ethics Statement

The studies involving human participants were reviewed and approved by CMO Region Arnhem/Nijmegen, Netherlands. The patients/participants provided their written informed consent to participate in this study.

## Author Contributions

PV, BJ, and MH collected the data. VT-P and MH performed the analysis. MB and MH coded the creativity data. MS, MB, EK, and MH designed the analysis. BF and MH were involved in designing and setting up the IMpACT and BIG studies. MS, VT-P, and MH wrote the paper with contributions from all the authors. All authors contributed to the article and approved the submitted version.

## Funding

MH was supported by a personal Veni grant from the Netherlands Organization for Scientific Research (NWO, grant number 91619115). BIG: This study used the BIG database, which was established in Nijmegen in 2007. This resource is now part of Cognomics, a joint initiative by researchers of the Donders Centre of Cognitive Neuroimaging, the Human Genetics and Cognitive Neuroscience Departments of the Radboud University Medical Centre, and the Max Planck Institute for Psycholinguistics. The Cognomics Initiative was supported by the participating departments and centres and by external grants, including grants from the Biobanking and Biomolecular Resources Research Infrastructure (Netherlands) (BBMRI-NL) and the Hersenstichting Nederland. In particular, the authors would also like to acknowledge grants supporting their work from the Netherlands Organization for Scientific Research (NWO), i.e. the NWO Brain & Cognition Excellence Program (grant 433-09- 229) and the Vici Innovation Program (grant 016–130-669 to BF). Additional support is received from the European Community's Seventh Framework Programme (FP7/2007 – 2013) under grant agreements n° 602805 (Aggressotype), n° 603016 (MATRICS), n° 602450 (IMAGEMEND), and n° 278948 (TACTICS), and from the European Community's Horizon 2020 Programme (H2020/2014 – 2020) under grant agreements n° 643051 (MiND) and n° 667302 (CoCA). IMpACT: We acknowledge funding from the Netherlands Organization for Scientific Research (NWO), the Vici Innovation Program (grant 016–130-669 to BF). The work was also supported by grant U54 EB020403 to the ENIGMA Consortium from the BD2K Initiative, a cross-NIH partnership, and by the European College of Neuropsychopharmacology (ECNP) Network “ADHD Across the Lifespan.”

## Conflict of Interest

BF has received educational speaking fees from Medice. The remaining authors declare that the research was conducted in the absence of any commercial or financial relationships that could be construed as a potential conflict of interest.

## Publisher's Note

All claims expressed in this article are solely those of the authors and do not necessarily represent those of their affiliated organizations, or those of the publisher, the editors and the reviewers. Any product that may be evaluated in this article, or claim that may be made by its manufacturer, is not guaranteed or endorsed by the publisher.
