# Supplementary material for: Characterizing Creative Thinking and Creative Achievements in Relation to Symptoms of Attention-Deficit/Hyperactivity Disorder and Autism Spectrum Disorder
Source: Front Psychiatry. 2022 Jul 1;13:909202. doi: 10.3389/fpsyt.2022.909202 (PMC9283685; doi:10.3389/fpsyt.2022.909202)
Supplement: Supplementary file 1 [file Table_1.DOCX]

**SUPPLEMENTS**

**Supplementary methods**

1. **Description of the BIG online testing waves**

Below you can find a visual representation of the time line of the online testing waves of the BIG/Cognomics study. On average there were on average 7 years between IBIG1 and IBIG2.1, and 1 year between IBIG2.1 & IBIG2.2

IBIG1

ADHD symptoms

IBIG2.1

ASD symptoms & CAQ

IBIG2.2

AUT & RAT

*Note: AUT= alternative uses task that measures divergent thinking, RAT= remote associations test that measures convergent thinking, CAQ= creative achievement task that measures creative achievements in different domains.*

1. **Description of the IMpACT2-NL sample**

The objective of the IMpACT2-NL study was to collect data from individuals with ADHD and controls on multiple levels (genes, brain, cognition, behaviour) to increase our understanding of adult ADHD. The study is part of a larger collaboration called the International Multicenter persistent ADHD CollaboraTion (IMpACT) with different nodes in Europe and the USA, https://www.impactadhdgenomics.com. The IMpACT2-NL study has a protocol that is an update from the original IMpACT-NL study (1,2). Participants were recruited via advertisements and the national patient organizations. All participants provided written informed consent before participating in the study and received monetary compensation for their participation. The study was approved by the local medical ethical committee.

Participants were included in the ADHD group if they had been diagnosed with ADHD by a clinician. To confirm the diagnosis and assess previous and current symptoms in all participants, we used the Diagnostic Interview for Adult ADHD (DIVA 2.0). Exclusion criteria for all participants were: (1) younger than 18 or older than 60 years of age, (2) neurological disorders, (3) psychosis or substance abuse in the last 6 months, (4) current major depression, (5) psycho-pharmaceutical therapy other than stimulants, (6) impairments of hearing, seeing and sensorimotor abilities. The study protocol consisted of two visits and an online test battery. During the first visit the DIVA interview was conducted by a trained researcher. The creativity tasks were part of the online test battery. The participants with ADHD who were on pharmacological treatment with stimulants (59%), were instructed to use their regular medication during the online testing.

1. **A list of the 18 ADHD DSM symptoms**

*Inattention*

1. Makes careless mistakes/lacks attention to detail
2. Difficulty sustaining attention
3. Does not seem to listen when spoken to directly
4. Fails to follow through on tasks and instructions
5. Exhibits poor organization
6. Avoids/dislikes tasks requiring sustained mental effort
7. Loses things necessary for tasks/activities
8. Easily distracted (including unrelated thoughts)
9. Is forgetful in daily activities

*Hyperactivity*

1. Fidgets with or taps hands or feet, squirms in seat
2. Leaves seat in situations when remaining seated is expected
3. Experiences feelings of restlessness
4. Has difficulty engaging in quiet, leisurely activities
5. Is “on-the-go” or acts as if “driven by a motor”
6. Talks excessively

*Impulsivity*

1. Blurts out answers
2. Has difficulty waiting their turn
3. Interrupts or intrudes on others

1. **A list of the items of the short autism questionnaire (AQ18)**

*Child behaviors*

*5. As a child, I was a late talker or I had other speech-related problems

*13. As a child, I often retreated to my own world or I rarely played with other children

*14. As a child, I moved in a rigid way or I tended to repeat certain movements

*16. As a child, I often took statements and jokes literally

18. As a child, I frequently became upset by sudden and unexpected changes

*Rigidity*

*7. People tell me that I keep going on and on about the same thing

*9. I often get so absorbed in one thing that I lose sight of other things

*10. It upsets me if my daily routine is disturbed

*11. I prefer to do things the same way over and over again

*Social difficulties (original ‘Social skills’ scale)*

*2. I find it hard to make new friends

3. I enjoy social occasions such as birthdays, receptions, and so on

*6. I don’t know how to keep a conversation going

*Attention to detail*

1. By looking at someone’s face, I find it easy to work out what he or she is thinking or feeling

4. I can quickly work out whether someone is fascinated by what I say

*6. I don’t know how to keep a conversation going

*Problems with Imagination (original ‘Imagination’ scale)*

8. I find making stories up easy

17. As a child, I enjoyed playing games involving pretending with other children

*Reversely scores item

Note: we renamed two of the original scales from (3) to better match the direction of the scales. For example, a high score on ‘social skills’ means less social skills and therefore we renamed the scale social difficulties.

1. **Example of one domain of the Creative Achievement Questionnaire.**

Music

__0. I have no training or recognized talent in this area.

__1. I play one or more musical instruments proficiently.

__2. I have played with a recognized orchestra or band.

__3. I have composed an original piece of music.

__4. My musical talent has been critiqued in a local publication.

__5. My composition has been recorded.

__6. Recordings of my composition have been sold publicly.

__7. My compositions have been critiqued in a national publication.

**Supplementary Tables**

**Supplementary Table 1.** Intra-rater reliability and agreement of the Alternative Uses Task in BIG/Cognomcs and the IMpACT2-NL sample. The raters were the same for both datasets (MB and MH).

|  | BIG/Cognomics | | IMpACT2-NL | |
| --- | --- | --- | --- | --- |
|  | Cohen’s Kappa (flexibility) | ICC  (originality) | Kappa (flexibility) | ICC  (originality) |
| Brick | .80, p < .0001 | .73, p < .0001 | .79, p <.0001 | .92, p < .0001 |
| Newspaper | .76, p < .0001 | .69, p < .0001 | .85, p < .0001 | .89, p < .0001 |
| Shoe | .85, p < .0001 | .78, p < .0001 | .73, p < .0001 | .94, p < .0001 |

*Note.* ICC= intraclass correlation coefficient. All agreements are in the range of good or excellent (4) (128).

**Supplementary Table 2.** Non-parametric correlations between creative performance measurements (AUT/RAT) and creative achievements (CAQ) in the BIG study (population-based sample).

|  |  | 1. | 2. | 3. | 4. | 5. | 6. | 7. | 8. |
| --- | --- | --- | --- | --- | --- | --- | --- | --- | --- |
| 1. RAT total | *r*  *p* |  |  |  |  |  |  |  |  |
| 2. AUT fluency | *r*  *p* | .181  .008 |  |  |  |  |  |  |  |
| 3. AUT flexibility | *r*  *p* | .269  .000 | .855  .000 |  |  |  |  |  |  |
| 4. AUT originality | *r*  *p* | .137  .045 | .348  .000 | .501  .000 |  |  |  |  |  |
| 5. CAQ total | *r*  *p* | .136  .049 | .167  .016 | .277  .000 | .180  .000 |  |  |  |  |
| 6. CAQ science | *r*  *p* | .139  .045 | .154  .026 | .218  .002 | .066  .341 | .764  .000 |  |  |  |
| 7. CAQ expressive | *r*  *p* | .035  .617 | .147  .034 | .247  .000 | .201  .004 | .637  .000 | .211  .000 |  |  |
| 8. CAQ performance | *r*  *p* | .150  .031 | .081  .243 | .197  .004 | .199  .004 | .559  .000 | .178  .000 | .218  .000 |  |

**Supplementary Table 3.** Correlation matrix of ADHD and ASD symptom domains and creative achievement scores in the BIG study (population-based sample).

|  |  | CAQ total | CAQ science | CAQ expressive | CAQ performance |
| --- | --- | --- | --- | --- | --- |
| ADHD | Inattention | *r* = .14, *p* = .002 | *r* = .07, *p* = .14 | *r* = .19, *p* < .0001 | *r* = .05, *p* = .31 |
|  | Hyperactivity impulsivity | *r* = .04, *p* = .37 | *r* = .03, *p* = .53 | *r* = .08, *p* = .10 | *r* = .01, *p* = .81 |
| ASD | Child behavior | *r* = .09, *p* = .04 | *r* = .13, *p* = .005 | *r* = .09, *p* = .06 | *r* = -.03, *p* = .48 |
|  | Rigidity | *r* = .02, *p* = .60 | *r* = .04, *p* = .34 | *r* = .03, *p* = .55 | *r* = -.04, *p* = .38 |
|  | Social difficulties | *r* = .01, *p* = .80 | *r* = .02, *p* = .71 | *r* = .02, *p* = .65 | *r* = - .05, *p* = .33 |
|  | Attention detail | *r* = .09, *p* = .05 | *r* = .14, *p* = .002 | *r* = .07, *p* = .12 | *r* = -.08, *p* = .09 |
|  | Problems with Imagination | *r* = -.23, *p* < .0001 | *r* = -.04, *p* = .39 | *r* = -.32, *p* < .0001 | *r* = -.20, *p* < .0001 |

**Supplementary Table 4.** Demographics and ADHD and creativity scores of the IMpACT2 study (case-control sample).

|  | **Cases**  **N=79** | **Controls**  **N=72** | **p-value for the group comparison** |
| --- | --- | --- | --- |
| Percentage male | 43% | 50% | 0.42 |
| Average age in years (SD) | 33.9 (10.1) | 35.4 (13.8) | 0.46 |
| Average ADHD symptoms* (SD) | 13.2 (3.0) | 1.3 (1.7) | <0.001 |
| Average AUT fluency (SD) | 8.5 (4.1) | 6.0 (4.0) | <0.001 |
| Average AUT flexibility (SD) | 6.0 (2.3) | 4.5 (2.5) | <0.001 |
| Average AUT originality (SD) | 2.0 (0.4) | 1.9 (0.5) | 0.03 |

Note: *ADHD symptoms were measured with the DIVA interview with a possible range of 0-18.

**Supplementary Table 5.** Effect of current stimulant medication use on divergent thinking in individuals with ADHD in the IMpACT2 study (case-control study)

| **Creativity measurement** | **Standardized beta** | **p-value** |
| --- | --- | --- |
| AUT fluency | 0.20 | 0.08 |
| AUT flexibility | 0.19 | 0.11 |
| AUT originality | -0.02 | 0.85 |

**Supplementary References**

1. Mostert JC, Hoogman M, Onnink AMH, van Rooij D, von Rhein D, van Hulzen KJE, et al. Similar subgroups based on cognitive performance parse heterogeneity in adults with ADHD and healthy controls. J Atten Disord. 2018;22(3):281–92.

2. Mostert JC, Onnink AMH, Klein M, Dammers J, Harneit A, Schulten T, et al. Cognitive heterogeneity in adult attention deficit/hyperactivity disorder: A systematic analysis of neuropsychological measurements. Eur Neuropsychopharmacol. 2015;25(11):2062–74. Available from: http://dx.doi.org/10.1016/j.euroneuro.2015.08.010

3. Bralten J, van Hulzen K, Martens M. B, Galesloot T E, Arias Vasquez A, Kiemeney A, Buitelaar J K, Muntjewerff J W, Franke B, Poelmans G. 2018. Autism spectrum disorders and autistic traits share genetics and biology. Mol Psychiatry, May;23(5):1205-1212.

3. Cicchetti DV, Sparrow SA. Developing criteria for establishing interrater reliability of specific items: applications to assessment of adaptive behavior. Am J Ment Defic. 1981;86(2):127-37.
